# Long‐term gynecological symptoms and ultrasound and reproductive outcomes after monofilament *vs* multifilament uterine closure at Cesarean delivery: secondary analysis of randomized controlled trial

**DOI:** 10.1002/uog.70365

**Published:** 2026-10-27

**Authors:** C. Ziello, A. M. Pacilio, L. Niccolini, S. Mola, C. Casella, A. Silvestri, L. Falco, A. Di Spiezio Sardo, G. Saccone, Collaborators, A. Bello, F. Di Napoli, C. Murolo, A. M. Merone

**Affiliations:** ^1^ Department of Public Health School of Medicine, University of Naples Federico II Naples Italy; ^2^ Department of Advanced Biomedical Sciences University of Naples Federico II Naples Italy; ^3^ Department of Neuroscience, Reproductive Science and Dentistry School of Medicine, University of Naples Federico II Naples Italy

**Keywords:** Cesarean delivery, Cesarean‐scar defect, isthmocele, postmenstrual spotting, suture material, uterine closure

## Abstract

**Objective:**

To evaluate long‐term gynecological symptoms and reproductive and ultrasound outcomes among women originally enrolled in a randomized controlled trial (RCT) comparing monofilament and multifilament sutures for uterine closure after Cesarean delivery.

**Methods:**

This was a secondary analysis of a RCT including women who underwent Cesarean delivery, who were originally assigned to monofilament or multifilament absorbable sutures for uterine closure. In this secondary analysis, participants were contacted 4–6 years after delivery and invited to a follow‐up evaluation, comprising a clinical interview and a voluntary transvaginal ultrasound examination. The primary outcome was the presence of postmenstrual spotting at long‐term follow‐up. Secondary outcomes were other gynecological symptoms, reproductive outcomes and ultrasound outcomes. Analyses were performed according to the original randomized allocation. Effect estimates are reported as relative risk (RR) with 95% CI.

**Results:**

Of the 300 women included in the original RCT, 117 (39.0%) were included in the long‐term follow‐up analysis, comprising 51 in the monofilament group and 66 in the multifilament group. There was no significant difference in the primary outcome of postmenstrual spotting between the monofilament and multifilament groups (23.5% *vs* 22.7%; RR, 1.04 (95% CI, 0.53–2.01)). Similarly, no significant differences were observed between groups for other gynecological symptoms. Ultrasound data were available for 21 women, of whom 13 had a Cesarean‐scar defect. The overall prevalence of Cesarean‐scar defects was similar between groups, although defects ≥ 4 mm were more frequent in the multifilament group (RR, 0.42 (95% CI, 0.18–0.97); *P* = 0.04). Most reproductive outcomes, including infertility and pregnancy outcomes among women who conceived, were comparable between groups. However, subsequent pregnancy occurred more frequently in the monofilament group (29.4% *vs* 12.1%; RR, 2.43 (95% CI, 1.12–5.27)).

**Conclusions:**

In this long‐term follow‐up of a RCT, monofilament and multifilament sutures for uterine closure were associated with similar long‐term gynecological symptoms. Differences observed in selected ultrasound and reproductive outcomes should be interpreted cautiously because of the limited sample size and exploratory nature of these analyses. Further studies are needed to better evaluate the potential impact of suture material on long‐term reproductive outcomes and sonographic Cesarean‐scar characteristics. © 2026 The Author(s). *Ultrasound in Obstetrics & Gynecology* published by John Wiley & Sons Ltd on behalf of International Society of Ultrasound in Obstetrics and Gynecology.

## INTRODUCTION

Cesarean delivery is one of the most commonly performed surgical procedures worldwide, with the rate increasing steadily over the past decades^1^. As the number of Cesarean deliveries performed has risen, long‐term complications related to Cesarean‐scar healing have gained increasing clinical relevance^2^, ^3^. Cesarean‐scar defects, also referred to as niches or isthmoceles, are characterized by a myometrial discontinuity at the site of a previous uterine incision. These defects have been associated with a range of gynecological symptoms, including abnormal uterine bleeding, particularly postmenstrual spotting, dysmenorrhea, chronic pelvic pain and dyspareunia^4^.

Several surgical factors at the time of Cesarean delivery have been proposed to influence Cesarean‐scar healing, including the technique of uterine closure, the use of locking or non‐locking sutures and the type of suture material^5^, ^6^, ^7^, ^8^, ^9^, ^10^. Among these, the type of suture material may play a role in Cesarean‐scar healing through differences in tissue reactivity, inflammatory response and absorption profile. Monofilament sutures are generally considered to be associated with reduced tissue drag and reduced inflammatory response compared with multifilament sutures^11^. However, whether these theoretical advantages translate into clinically meaningful differences in Cesarean‐scar healing remains unclear^8^.

In a recent randomized controlled trial (RCT) comparing monofilament and multifilament absorbable sutures for uterine closure at the time of Cesarean delivery, no significant difference was observed in the incidence of Cesarean‐scar defects at 6 months after delivery, although differences in specific ultrasound characteristics of the Cesarean scar were noted^8^. However, short‐term ultrasound findings may not fully reflect the long‐term clinical relevance of Cesarean‐scar healing. In particular, the relationship between early postoperative Cesarean‐scar characteristics and long‐term gynecological symptoms remains poorly understood. In addition, data on long‐term ultrasound outcomes, including persistence or progression of Cesarean‐scar defects and changes in residual myometrial thickness (RMT), are limited.

Thus, the aim of this secondary analysis was to evaluate the long‐term gynecological symptoms and reproductive and ultrasound outcomes among women originally enrolled in a RCT comparing monofilament and multifilament sutures for uterine closure after Cesarean delivery.

## METHODS

### Study design and population

This was a secondary analysis of a RCT comparing monofilament with multifilament absorbable sutures for uterine closure at the time of Cesarean delivery^8^. The parent trial enrolled women with a singleton pregnancy at term undergoing either primary or secondary Cesarean delivery at the University of Naples Federico II, Naples, Italy, between February 2020 and August 2022. Short‐term ultrasound outcomes, including sonographic characteristics of the Cesarean scar, and the presence of Cesarean‐scar defects, were evaluated 6 months after delivery. In the present study, all women originally randomized in the trial were considered eligible for long‐term follow‐up, and were contacted and invited to participate in a single follow‐up visit. Exclusion criteria for the follow‐up evaluation included inability to be contacted and refusal to participate. This trial was approved by the local Institutional Review Board of the University of Naples Federico II on 2 July 2019 and registered at ClinicalTrials.gov (registration no.: NCT04073264).

### Follow‐up procedures

Participants were contacted by telephone or electronic communication 4–6 years after delivery, and were invited to attend a dedicated follow‐up visit. Multiple contact attempts were performed as necessary. Reasons for non‐participation were recorded when available. At the follow‐up visit, all participants underwent a structured clinical interview. Participants were also invited to undergo a transvaginal ultrasound examination; however, participation in the ultrasound assessment was voluntary and depended on participant availability. The original trial was conducted as an open‐label study, and no formal blinding was used during the long‐term follow‐up.

### Clinical assessment

Gynecological symptoms were assessed using a standardized questionnaire completed during a clinical interview at the time of the follow‐up visit. The following symptoms were recorded as present or absent: abnormal uterine bleeding, postmenstrual spotting, chronic pelvic pain, dysuria, dyspareunia and urinary incontinence. Symptoms were self‐reported by the participants. Postmenstrual spotting was recorded as a questionnaire item separate to abnormal uterine bleeding. No validated symptom‐severity scale was applied, and symptoms were analyzed as binary variables (present or absent). Information on reproductive history after the index Cesarean delivery was also collected, including subsequent pregnancies and their outcomes, when available.

### Ultrasound evaluation

The Cesarean scar was evaluated by transvaginal ultrasound examination, in participants who agreed to the scan. Cesarean‐scar defect was defined as an anechoic or hypoechogenic defect at the site of the previous uterine incision communicating with the endometrial cavity, with a depth of at least 2.0 mm^8^, ^12^. For each participant, the following measurements were obtained in the sagittal plane: depth and width of the Cesarean‐scar defect, when present, and minimum RMT at the level of the Cesarean scar. Ultrasound scans were performed during the secretory phase of the menstrual cycle when possible in order to minimize variability related to endometrial thickness and intracavitary fluid^13^.

### Primary and secondary outcomes

The primary outcome of this secondary analysis was the presence of postmenstrual spotting at long‐term follow‐up. Secondary outcomes included: the presence of other gynecological symptoms, specifically abnormal uterine bleeding, chronic pelvic pain, dysuria, dyspareunia and urinary incontinence; reproductive outcomes, specifically occurrence of subsequent pregnancy and pregnancy complications, infertility (defined as failure to achieve a spontaneous pregnancy after at least 12 months of attempting) and mode of delivery of subsequent pregnancies; and ultrasound outcomes, specifically the presence of a Cesarean‐scar defect^12^, depth of the Cesarean‐scar defect and minimum RMT at the level of the Cesarean scar, when available.

### Statistical analysis

All analysis was performed according to the original randomized allocation. For the primary outcome, the proportion of women reporting postmenstrual spotting at long‐term follow‐up was compared between groups using the chi‐square test. Effect estimates are reported as relative risk (RR) with 95% CI. Secondary binary outcomes were analyzed similarly, using the chi‐square test or Fisher's exact test, with results reported as RR (95% CI). For binary outcomes with zero events in one or more cells, a Haldane–Anscombe continuity correction was applied. Continuous variables are summarized as mean ± SD or median (interquartile range), as appropriate based on data distribution, and categorical variables are given as *n* (%). Comparisons between groups were performed using Student's *t*‐test or the Mann–Whitney *U*‐test. Statistical analysis was performed using SPSS version 26 (IBM Corp., Armonk, NY, USA). A two‐sided *P*‐value < 0.05 was considered statistically significant.

## RESULTS

### Trial population and follow‐up

Of the 300 women randomized in the original RCT, 117 (39.0%) were successfully contacted and included in the long‐term follow‐up analysis. Of these, 21 (17.9%) also underwent a follow‐up transvaginal ultrasound scan. Inclusion in the long‐term follow‐up analysis differed by randomized group: 51/151 (33.8%) were included from the monofilament group and 66/149 (44.3%) from the multifilament group. The median duration of follow‐up was 5.5 (interquartile range, 5.3–5.8) years. Baseline demographic and clinical characteristics of the women included in the secondary analysis were generally similar between the two treatment groups, although simple continuous suturing was more frequent in the monofilament group than in the multifilament group (96.1% *vs* 75.8%; *P* = 0.02) (Table 1). A comparison of baseline demographic and clinical characteristics between women included and those not included in the long‐term follow‐up analysis is shown in Table S1.

**Table 1:** Baseline demographic and clinical characteristics of women included in secondary analysis of randomized controlled trial comparing mono‐ and multifilament sutures for uterine closure after Cesarean delivery, according to suture material

Characteristic	Monofilament (*n* = 51)	Multifilament (*n* = 66)	*P*
Age (years)	33.2 ± 5.0	33.3 ± 5.6	0.92
BMI (kg/m^2^)	25.8 ± 3.7	27.0 ± 4.6	0.12
White ethnicity	51 (100)	64 (97.0)	0.31
Nulliparous	32 (62.7)	40 (60.6)	0.81
Prior Cesarean delivery	17 (33.3)	29 (43.9)	0.25
Planned Cesarean delivery of the index pregnancy	26 (51.0)	35 (53.0)	0.83
Single layer suture	39 (76.5)	53 (80.3)	0.62
Simple continuous suture	49 (96.1)	50 (75.8)	0.02
Locked continuous suture	49/49 (100)	47/50 (94.0)	0.13

Data are presented as mean ± SD, *n* (%) or *n*/*N* (%). BMI, body mass index.

### Gynecological symptoms

At long‐term follow‐up, there was no significant difference between groups in the primary outcome of postmenstrual spotting, which was reported in 12/51 (23.5%) women in the monofilament group and 15/66 (22.7%) women in the multifilament group (RR, 1.04 (95% CI, 0.53–2.01)) (Table 2).

Similarly, no significant differences were observed between groups for other gynecological symptoms. Abnormal uterine bleeding was reported in 18/51 (35.3%) women in the monofilament group and 18/66 (27.3%) women in the multifilament group (RR, 1.29 (95% CI, 0.75–2.22)). Chronic pelvic pain was reported in 8/51 (15.7%) women in the monofilament group and 11/66 (16.7%) women in the multifilament group (RR, 0.94 (95% CI, 0.41–2.17)). Dyspareunia was reported in 5/51 (9.8%) women in the monofilament group and 8/66 (12.1%) women in the multifilament group (RR, 0.81 (95% CI, 0.28–2.33)). Dysuria and urinary incontinence were infrequent and distributed similarly between the groups.

**Table 2:** Primary and secondary outcomes of women included in secondary analysis of randomized controlled trial comparing mono‐ and multifilament sutures for uterine closure after Cesarean delivery, according to suture material

Outcome	Monofilament (*n* = 51)	Multifilament (*n* = 66)	RR (95% CI)	*P*
Gynecological symptoms				
Abnormal uterine bleeding	18 (35.3)	18 (27.3)	1.29 (0.75–2.22)	0.35
Postmenstrual spotting	12 (23.5)	15 (22.7)	1.04 (0.53–2.01)	0.92
Chronic pelvic pain	8 (15.7)	11 (16.7)	0.94 (0.41–2.17)	0.89
Dysuria	3 (5.9)	3 (4.5)	1.29 (0.27–6.15)	0.75
Dyspareunia	5 (9.8)	8 (12.1)	0.81 (0.28–2.33)	0.69
Urinary incontinence	3 (5.9)	6 (9.1)	0.65 (0.17–2.46)	0.52
Reproductive outcomes				
Attempted to conceive	13 (25.5)	10 (15.2)	1.68 (0.80–3.52)	0.17
Infertility	0/13 (0)	2/10 (20.0)	0.16 (0.01–2.95)	0.38
Unintended pregnancy	2 (3.9)	0 (0)	6.44 (0.32–131.31)	0.23
Occurrence of subsequent pregnancy	15 (29.4)	8 (12.1)	2.43 (1.12–5.27)	0.03
Spontaneous miscarriage	1/15 (6.7)	0/8 (0)	1.69 (0.08–37.26)	0.40
Live birth	14/15 (93.3)	8/8 (100)	0.96 (0.77–1.20)	0.72
Cesarean delivery	14/14 (100)	8/8 (100)	1.00 (0.83–1.20)	0.99
Pregnancy complications	2/14* (14.3)	1/8† (12.5)	1.14 (0.12–10.71)	0.91
Ultrasound outcomes				
Cesarean‐scar defect	8/13 (61.5)	5/8 (62.5)	0.98 (0.50–1.96)	0.96
Cesarean‐scar defect ≥ 4 mm	3/8 (37.5)	5/5 (100)	0.42 (0.18–0.97)	0.04
Minimum RMT < 3 mm	1/13 (7.7)	2/8 (25.0)	0.31 (0.03–2.87)	0.30

Data are presented as *n* (%) or *n*/*N* (%), unless stated otherwise.

* One case of intrauterine growth restriction and one case of gestational diabetes mellitus.

† One case of placenta previa. RMT, residual myometrial thickness; RR, relative risk.

### Reproductive outcomes

Among women included in the secondary analysis, 13/51 (25.5%) in the monofilament group and 10/66 (15.2%) in the multifilament group reported that they attempted to conceive. Infertility was not reported in any women in the monofilament group but in 2/10 (20.0%) women in the multifilament group.

A subsequent pregnancy after the index pregnancy occurred in 15/51 (29.4%) women in the monofilament group and 8/66 (12.1%) women in the multifilament group (RR, 2.43 (95% CI, 1.12–5.27); *P* = 0.03). Among women who had a subsequent pregnancy, spontaneous miscarriage occurred only in 1/15 (6.7%) women in the monofilament group. Live birth occurred in the remaining 14/15 (93.3%) subsequent pregnancies in the monofilament group and in all eight (100%) pregnancies in the multifilament group (RR, 0.96 (95% CI, 0.77–1.20)). All deliveries in both groups were achieved via Cesarean section. No women underwent *in‐vitro* fertilization during follow‐up, and all subsequent pregnancies were normally sited, with no ectopic or Cesarean‐scar pregnancies reported. Obstetric complications were infrequent and similar between groups (Table 2).

### Sonographic Cesarean‐scar characteristics

Ultrasound data at long‐term follow‐up were available for a total of 21 women, of whom 19/21 (90.5%) underwent ultrasound examination during the secretory phase of the menstrual cycle. Overall, 13 patients in the monofilament group and eight in the multifilament group underwent ultrasound examination. In the monofilament group, 8/13 (61.5%) women presented with an ultrasonographically detectable Cesarean‐scar defect. Among these, three (37.5%) patients exhibited a Cesarean‐scar defect with a depth of ≥ 4 mm. None of the patients with a Cesarean‐scar defect showed a minimum RMT < 3 mm. However, one patient without evidence of a Cesarean‐scar defect had a reduced minimum RMT of 2.8 mm. In the multifilament group, 5/8 (62.5%) women had a Cesarean‐scar defect detected on ultrasound examination. Notably, all these defects had a depth of ≥ 4 mm. Additionally, two patients in this group had a minimum RMT of 2.8 mm. Overall, the prevalence of Cesarean‐scar defect was comparable between the two groups, although larger defects (≥ 4 mm) appeared significantly more frequently in the multifilament group (RR, 0.42 (95% CI, 0.18–0.97); *P =* 0.04) (Table 2).

## DISCUSSION

### Main findings

In this secondary analysis of a RCT comparing monofilament and multifilament sutures for uterine closure after Cesarean delivery^8^, we found no significant differences in long‐term gynecological symptoms between the two groups. In particular, the rate of postmenstrual spotting (the primary outcome) was similar between women who received monofilament sutures and those who received multifilament sutures. Reproductive outcomes and ultrasound outcomes were also largely comparable between the groups. A higher rate of subsequent pregnancy was observed in the monofilament group; however, this finding should be interpreted with substantial caution given the exploratory nature of the analysis, the small number of women who attempted to conceive, the low follow‐up rate and the possibility of residual confounding related to differences in reproductive intentions and obstetric history. Therefore, no causal relationship between suture material and subsequent fertility can be inferred from these data.

The results of this study are consistent with those of the parent RCT, which showed no significant difference between monofilament and multifilament sutures in terms of the incidence of Cesarean‐scar defect, minimum RMT and short‐term gynecological symptoms at 6 months after delivery^8^. Taken together, these findings suggest that suture material used for uterine closure after Cesarean delivery may not have a clinically meaningful impact on either short‐term scar healing or long‐term gynecological symptoms.

### Strengths and limitations

This study has several strengths. First, it represents a long‐term follow‐up of a RCT, allowing the evaluation of clinically relevant outcomes several years after Cesarean delivery. Second, the original randomized design reduced the risk of selection bias when comparing the two suture materials. Third, detailed information on gynecological symptoms and reproductive outcomes was systematically collected.

However, several limitations should be acknowledged. The most important is the relatively low follow‐up rate, with only 39% of the original cohort included in the long‐term secondary analysis, and an imbalance in the follow‐up rate between treatment groups. This may have introduced selection bias and limited the generalizability of the findings. Second, the study was not powered for the assessment of long‐term outcomes, and the sample size of the follow‐up cohort may have been insufficient to detect small but clinically meaningful differences between the groups. Third, analyses related to reproductive outcomes were based on a small number of events and should therefore be considered exploratory and hypothesis generating. Fourth, ultrasound data at long‐term follow‐up were not available for all participants, and therefore the relationship between changes in Cesarean‐scar morphology over time and long‐term symptoms could not be assessed. Indeed, ultrasound follow‐up data were available for only 21 women (17.9% of the follow‐up cohort), as ultrasound examination was an optional component of the follow‐up assessment and participation depended on individual availability. Consequently, the ultrasound findings should be interpreted with caution given the small sample size and potential selection bias. The relatively low follow‐up rate may also have introduced attrition bias. Although comparison of baseline characteristics between the women included and those not included in the follow‐up cohort provides useful information regarding cohort representativeness (Table S1), the possibility of residual bias related to unmeasured factors cannot be excluded. Finally, although treatment allocation was randomized, some surgical characteristics that were not specifically randomized, including single‐layer *vs* double‐layer uterine closure and locking *vs* non‐locking sutures (Table 1), may still have influenced outcomes and should therefore be considered when interpreting these findings.

### Implications

Cesarean‐scar defects are being increasingly recognized as a relevant long‐term consequence of Cesarean delivery, with potential implications such as abnormal uterine bleeding, pelvic pain and complications in subsequent pregnancies^14^. The pathophysiology of Cesarean‐scar defect formation is multifactorial and may include surgical technique, patient characteristics and healing processes^15^. Despite theoretical differences in tissue handling and healing between monofilament and multifilament sutures, our findings suggest that suture material alone is unlikely to play a major role in determining long‐term clinical outcomes. These findings are broadly consistent with a previous systematic review and meta‐analysis of randomized controlled trials, which found insufficient evidence to recommend one type of suture material over another for uterine closure at Cesarean delivery^7^.

From a clinical perspective, these results support the concept that factors other than suture material, such as surgical technique, anatomical factors and patient‐related characteristics, may be more relevant determinants of long‐term Cesarean‐scar outcomes^16^. Future research should focus on identifying modifiable surgical factors and on understanding the relationship between early ultrasound findings and long‐term clinical symptoms.

### Conclusions

In this long‐term follow‐up of a RCT, the use of monofilament *vs* multifilament sutures for uterine closure after Cesarean delivery was not associated with differences in long‐term gynecological symptoms. Findings regarding reproductive outcomes should be considered exploratory and hypothesis generating. Further studies are needed to better evaluate the potential impact of suture material on long‐term reproductive outcomes and sonographic Cesarean‐scar characteristics. These findings, together with those of the parent trial obtained at 6 months after Cesarean delivery, suggest that suture material may not have a clinically meaningful impact on Cesarean‐scar healing and its long‐term consequences.

### Collaborators

**A. Bello**, Department of Public Health, School of Medicine, University of Naples Federico II, Naples, Italy

**F. Di Napoli**, Department of Public Health, School of Medicine, University of Naples Federico II, Naples, Italy

**C. Murolo**, Department of Public Health, School of Medicine, University of Naples Federico II, Naples, Italy

**A. M. Merone**, Department of Public Health, School of Medicine, University of Naples Federico II, Naples, Italy

## Supporting information


**Table S1** Baseline characteristics of women included and women not included in the long‐term follow‐up analysis.

## Data Availability

The data that support the findings of this study are available on request from the corresponding author. The data are not publicly available due to privacy or ethical restrictions.
