# Supplementary material for: Long‐term gynecological symptoms and ultrasound and reproductive outcomes after monofilament vs multifilament uterine closure at Cesarean delivery: secondary analysis of randomized controlled trial
Source: Ultrasound Obstet Gynecol. 2026 Oct 27;68(5):714–9. doi: 10.1002/uog.70365 (PMC13730430; doi:10.1002/uog.70365)
Supplement: Supplementary file 1 [file UOG-68-714-s001.docx]

**SUPPLEMENTARY TABLE**

**Supplementary Table** **1**. Baseline characteristics of women included and not included in the long-term follow-up analysis

|  | **Included**  **N = 117** | **Not included**  **N = 183** | **p-value** |
| --- | --- | --- | --- |
| **Age**  **mean ± SD (years)** | 33.3±5.3 | 32.7±5.5 | 0.35 |
| **Allocation group**  **Monofilament**  **n (%)**  **Multifilament**  **n (%)** | 51 (43.6%)  66 (56.4%) | 100 (54.6%)  83 (45.4%) | 0.06 |
| **Prior cesarean delivery**  **n (%)** | 46 (39.3%) | 63 (34.4%) | 0.39 |
| **Planned cesarean delivery**  **n (%)** | 61 (52.1%) | 88 (48.1%) | 0.49 |
| **Single layer**  **n (%)** | 92 (78.6%) | 127 (69.4%) | 0.07 |
| **BMI**  **mean ± SD** **(Kg/m^2^)** | 26.5±4.2 | 26.7±5.0 | 0.71 |

*Data are presented as n (%) or as mean ± standard deviation (SD)*

*BMI, body mass index.*
